# MEALTIME BEHAVIORS IN NURSING HOME RESIDENTS WITH DEMENTIA ARE ASSOCIATED WITH RESIDENT-INITIATED FOOD INTAKE

**DOI:** 10.1093/geroni/igae098.2277

**Published:** 2024-12-31

**Authors:** Heather Suh, Kyuri Lee, Wen Liu

**Affiliations:** University of Iowa, Iowa City, Iowa, United States; University of Iowa, Iowa City, Iowa, United States; University of Iowa, Iowa City, Iowa, United States

## Abstract

Nursing home (NH) residents with dementia experience challenging behaviors that interfere with their engagement, eating performance, and food intake during mealtime. Supporting resident-initiated food intake, defined as how well resident initiate and complete attempts of food intake with or without staff assistance, is a critical indicator of resident engagement and independence. Understanding the association between resident mealtime behaviors and resident-initiated food intake attempts informs future mealtime interventions. In a pilot trial for the OPTIMAL (Optimizing Mealtime) study, NH resident mealtime behaviors and interactions with direct care staff were observed and characterized. A total of 197 videotaped observations of full-meal resident-staff interactions were coded from 12 residents (age 71.9±7.5 years; 91.7% male; 91.7% white; 41.7% married). The average mealtime durations at breakfast, lunch, and dinner were 36.4 ± 20.6, 39.4 ± 16.7, and 39.7 ± 17.0 minutes, respectively. Out of 10,092 behaviors coded using the refined Cue Utilization and Engagement in Dementia (CUED) coding scheme, resident behaviors were categorized as positive/neutral behaviors (18.2%), chewing/swallowing difficulties (47.5%), functional impairment (13.3%), and resistive behaviors (21.0%). The majority of resident behaviors occurred at lunch (36.9%) and dinner (36.3%), with slightly fewer behaviors at breakfast (26.8%). Proportion of resident-initiated food intake attempts (0.51 ± 1.4, number of resident-initiated food intake attempts divided by total number of food intake attempts) was significantly associated with chewing/swallowing difficulties (b= -1.91, p=0.014), controlling for resident age and cognitive score. Addressing resident chewing/swallowing difficulties has the potential to improve resident-initiated food intake attempts to enhance resident mealtime engagement and independence.

